# Glucose metabolism involved in PD-L1-mediated immune escape in the malignant kidney tumour microenvironment

**DOI:** 10.1038/s41420-021-00401-7

**Published:** 2021-01-18

**Authors:** Yongbo Yu, Ye Liang, Dan Li, Liping Wang, Zhijuan Liang, Yuanbin Chen, Guofeng Ma, Hui Wu, Wei Jiao, Haitao Niu

**Affiliations:** 1 Department of Urology, The Affiliated Hospital of Qingdao University, Qingdao, 266003 China; 2 Key Laboratory, Department of Urology and Andrology, The Affiliated Hospital of Qingdao University, Qingdao, 266003 China

**Keywords:** Experimental models of disease, Drug development

## Abstract

Programmed death receptor-ligand 1 (PD-L1) plays a crucial role in immune evasion by tumour cells. Most tumour cells exhibit energy dependency and acquire energy from glycolysis. However, the relationship between glucose metabolism and PD-L1 expression remains unclear. In this study, changes in PD-L1 expression in renal carcinoma cells were evaluated during glucose deficiency and recovery, and PD-L1 could inversely regulate glycolysis. In addition, the possible signalling pathways activated by a low level of glucose to regulate PD-L1 were tested experimentally. The results showed that glucose deficiency could upregulate PD-L1 expression in two renal cancer cell lines, 786-O and OS-RC-2. Although the native levels of PD-L1 differed in the two cell lines, the upregulated PD-L1 expression was repristinated after glucose recovery. Moreover, epidermal growth factor receptor (EGFR) expression was upregulated in both cell lines with glucose deficiency. The use of an EGFR inhibitor reversed the upregulation of PD-L1 expression induced by glucose deficiency and inhibited the phosphorylation of extracellular regulated protein kinases 1 and 2 (ERK1/2). EGFR activated by epidermal growth factor (EGF) induced PD-L1 expression and ERK1/2 phosphorylation. Furthermore, an ERK1/2 inhibitor inhibited the phosphorylation of c-Jun and decreased the elevated PD-L1 expression induced by glucose deficiency. In addition, this study also showed that 6-phosphofructo-2-kinase/fructose-2,6-bisphosphatase 3 (PFK-2/FBPase 3 or PFKFB3) mediated upregulation of the level of glycolysis to improve the adverse environment through PD-L1 induction. Therefore, glucose metabolism can regulate the expression of PD-L1 through the EGFR/ERK/c-Jun pathway in renal cancer, and elevated PD-L1 can also regulate glycolysis by improving the expression of PFKFB3. The findings of this study could provide a new multiple target treatment for renal cell carcinoma (RCC) therapy.

## Introduction

Renal cell carcinoma (RCC) is one of the most lethal types of urological cancer, accounting for approximately 4% of malignant tumours and approximately 3% of the mortality related to malignant tumours^1^, but the prevalence of RCC has been increasing by approximately 2–4% every year for the last two decades around the globe^2^. Thereinto, clear cell renal cell carcinoma (ccRCC), the most common histological subtype, accounts for 70–80% of all RCC cases^2^. Immunotherapy with cytokines is a standard treatment for RCC patients. Unfortunately, only 5–20% of patients respond to this treatment. Tumour-induced anergy may in part explain the low response rate^3^. However, the mechanisms of immune dysfunction in cancer patients remain unclear. Under pathological conditions such as cancer, programmed death receptor-ligand 1 (PD-L1) expression is often upregulated in tumour cells, and PD-L1 binds to programmed cell death protein 1 (PD-1) on T cells, resulting in potent immune suppression and tumour immune escape^4–9^. Therefore, it has become very important to reduce the expression of PD-L1 or prevent the binding of PD-L1 to PD-1. PD-1/PD-L1 inhibitors have been applied in melanoma, non-small cell lung cancer (NSCLC)^10^^,^^11^ and renal cancer^12^, and have achieved certain effects, but their broader applicability is not extensive. As a consequence, we tried to find the pathway underlying the high expression of PD-L1, which could be combined with an intervention focused on a target in that pathway to reduce the expression of PD-L1 to suppress tumour immune escape^13^. Thus, the expression of PD-L1 on tumours strongly correlates with the survival of cancer patients.

Cancer cells, especially cells in metastatic tumour, are characterised by boosted glucose uptake and lactate production, and reduced respiration, even under aerobic conditions, which is known as the Warburg effect^14^. The Warburg effect describes the increased utilisation of glycolysis rather than oxidative phosphorylation by tumour cells to meet their energy requirements under physiological oxygen conditions. The Warburg effect has strong associations with invasion status, clinical stage, cancer prognosis and tumour drug resistance. The tumour microenvironment is characterised by hypoxia (low oxygen concentration) and has the potential to inhibit tumour cell differentiation^15^. In addition, the excessive lactate produced by glycolysis creates an acidic tumour microenvironment that promotes the migration and invasion of tumour cells^16^. The underlying molecular mechanisms and adaptive significance of this universal feature of malignant tumour cells have not been fully clarified in the eight decades since Warburg’s pioneering studies were performed^15^. The Warburg effect has been shown to be present in glucose metabolism in renal cancer^17^. The isozyme 6-Phosphofructo-2-kinase/fructose-2,6-bisphosphatase-3 (PFKFB3), which can activate 6-phosphofructo-1-kinase (PFK-1) into allostery, is a key regulator of glycolytic process. To date, four different genes coding different isozymes (PFKFB1–4) have been identified^18–21^. These isoenzymes differ in many aspects, such as the tissue distribution, kinetic and regulatory properties. The PFKFB3 isozyme has the highest kinase-to-phosphatase activity ratio thereby maintaining elevatedsfructose-2,6-bisphosphatase (F-2,6-P2) levels, which in turn sustain high glycolytic rates^22,23^. Significantly, this subtype has been found in several malignant tumour cell lines with high proliferative rates that require elevated activity of this enzyme to synthesise the precursor of purine and pyrimidine, 5-phosphoribose-1 pyrophosphate^22,24,25^. Alexander Minchenko’s study confirmed hypoxia-inducible factor-1 (HIF-1α)-mediated expression of the PFKFB3 gene^25^. Therefore, HIF-1α can regulate glycolysis through its target genes PFKFB3 and lactate dehydrogenase A (LDHA)^26,27^.

Epidermal growth factor receptor (EGFR), a glycoprotein belonging to the tyrosine kinase receptor family, has been implicated in the development, progression and severity of several human cancers, and is an attractive target for therapeutic intervention^28^. Glycolysis can strongly regulate EGFR expression^29^. In addition, the correlation between EGFR mutation and PD-L1 expression has been reported in non-small cell carcinoma^30^. It is known that phosphorylation of c-Jun at two serine residues (ser-63 and ser-73) results in c-Jun activation^31^. Mitogen-activated protein kinases (MAPKs), also known as extracellular regulated protein kinases (ERKs), have been proposed to play a role in this phosphorylation^32^, which is involved in signal transduction pathways triggered by EGFR in NSCLC.

In a preliminary experiment, we found that the protein and mRNA levels of PD-L1 were significantly upregulated under low-glucose conditions, and within 24 h, the lower the glucose concentration was, the higher the upregulation of PD-L1 expression. After discovering this phenomenon, we decided to investigate the mechanism underlying the upregulation of PD-L1 expression under low-glucose conditions. In this study, the regulatory relationship between glycolysis and PD-L1 was investigated, and the signalling pathways involved were detected. The results could provide a theoretical basis for the combination of targeted drugs for renal cancer treatment.

## Materials and methods

### Cell lines and reagents

The human renal cancer cell lines 786-O and OS-RC-2 were purchased from the Cell Bank of the Chinese Academy of Sciences (Shanghai, China). The two cell lines were also authenticated by VivaCell Biosciences Ltd. (Shanghai, China). Materials for cell culture, including fetal bovine serum (FBS; Gibco, Grand Island, NY, USA), Roswell Park Memorial Institute (RPMI) 1640 medium (MedChem Express, NJ, USA), penicillin and streptomycin (P/S) and trypsin were purchased from Gibco (Grand Island, NY, USA). Cells were cultured in 89% RPMI 1640 medium supplemented with 1% P/S and 10% FBS. Reagents for stimulating T cells included phorbol 12-myristate 13-acetate (PMA; MedChem Express, NJ, USA) and ionomycin (Iono; Solarbio, Beijing, China); 3-4,5-dimethylthiazol-2-yl-2,5 diphenyl tetrazolium bromide (MTT) was purchased from Sigma-Aldrich. All the cell lines were cultured in RPMI 1640 medium (glucose: 2000 mg/L) supplemented with 10% FBS and antibiotics (100 U/mL streptomycin and 100 U/mL penicillin) and maintained at 37 °C in a humidified 5% CO_2_ atmosphere. A mycoplasma stain assay kit (Beyotime, Haimen, China) was used for mycoplasma testing to rule out the possibility of cryptic contamination. Low-level glucose for 786-O cultivation medium was 1/4 (for OSRC-2: 1/16) normal RPMI 1640 medium (glucose: 2000 mg/L) and 3/4 (for OSRC-2: 15/16) glucose-free RPMI 1640 medium supplemented with 10% FBS and antibiotics (100 U/mL streptomycin and 100 U/mL penicillin). All other chemicals and reagents used in the study were reagent grade.

### The co-culture system of lymphocytes and tumour cells

Human peripheral T cells obtained from blood samples of healthy volunteers were processed with human lymphocyte separation medium (Solarbio, Beijing, China) for the preparation of peripheral blood mononuclear cells (PBMCs). For this, 5 mL fresh peripheral blood was mixed with normal saline at 1:1, and carefully added to the liquid surface of 10 mL of cell separation solution. After a series of layering, washing and centrifugation steps according to the manufacturer’s instruction, we obtained the isolated PBMCs. Then, we cultured the isolated PBMCs in a culture flask precoated with RetroNectin (Takara, Kusatsu, Japan) and anti-human CD3 antibody (Bio-Tool, Beijing, China), and added IFN-γ (Novus, Littleton, CO, USA) and interleukin-2 (PeproTech, Rocky Hill, NJ, USA) to the RPMI 1640 culture medium to activate and expand the CD3+ T cells^33^.

Then, 786-O and OS-RC-2 cells were cultured in different intervention groups in 24-well plates and normal control medium for 24 h, and the groups undergoing glucose deficiency that were simultaneously treated with PX-478, YC-1 or PFK-015 were compared. Then, the culture medium of the above groups was replaced with fresh normal medium, and the cells were cocultured with CD3+ T cells (3*10^5^). After that, different tumour cell groups were cultured with CD3+ T cells in fresh culture medium and the supernatants were analysed for IFN-γ production.

### Flow cytometry

The same PBMCs derived from healthy volunteers were cultured in RPMI 1640 medium, CD3 cells were activated and expanded with the same method given above. After culturing for 5 days, we used an anti-CD3 fluorescein isothiocyanate (FITC)-conjugated antibody (Thermo Fisher Scientific, Waltham, MA, USA); mouse IgG2a kappa isotype control, FITC (Thermo Fisher Scientific, clone eBM2a) and FACSCalibur (BD Biosciences, Franklin Lakes, NJ, USA) to assess the purity of the T cells.

### Cell culture and experimental groups

First, 786-O and OS-RC-2 cells were seeded in 24-well plates with RPMI 1640 culture medium (glu+: 2000 mg/L) and maintained for 24 h in a humidified incubator at 37 °C with 5% CO_2_. Then, the culture medium of the experimental group undergoing glucose deficiency was replaced with glucose-deficiency medium (glu−: 500 mg/L in 786-O and glu−: 125 mg/L in OS-RC-2), and the culture medium of the control group was replaced with fresh normal culture medium (glu+: 2000 mg/L). For the glucose recovery group, the cells were cultured with normal medium after glucose deficiency for 24 h.

### Lactic acid (LD) detection kit

LD was detected with an LD detection kit (Jiancheng Bioengineering Institute, Nanning, China). This assay is based on NAD+ ating as a hydrogen acceptor; LDH catalyses LD dehydrogenation to produce pyruvic acid, converting NAD+ into NADH. This conversion is mediated by a PMS hydrogen reducing NBT to create a purple colour, and the absorbance at 530 nm has a linear relationship with the LD concentration.

Reagent 2 (the enzyme storage solution) and Reagent 1 (enzyme diluent) were mixed at a volume ratio of 1:100, and the enzyme working solution was prepared immediately prior to use. The reagent and powder were added to the three-liquid Reagent 1 bottle at a 1:4 ratio, the bottle was shaken to dissolve the powder, and the liquid trace was moved to a small centrifuge tube. The centrifuge tube was repeatedly inverted, which allowed the liquid mix well. This process was repeated 2~3 times to mix the two reagents to create the chromogenic agent. Then, a blank group and standard were set up, and the sample group was evaluated in 96-well plates. In total, 20 µL of substrate, 1 mL of enzyme working solution and the chromogenic agent (200 µL were mixed and incubated in a 37 °C water bath after 10 min. Then, 2 mL of termination liquid was added to each well. After fully mixing, the absorbance values of all tubes were measured at a wavelength of 530 nm and light diameter of 1 cm.

### Inhibitor and stimulant treatments

To investigate the signalling pathways involved in the PD-L1 regulation mediated by glucose, the following inhibitors and stimulants were used. Recombinant human epidermal growth factor (EGF; Novus, USA) was used as a stimulant; the EGFR tyrosine kinase inhibitor (TKI) gefitinib and the ERK1/2 inhibitor U0126 (Monmouth Junction, NJ, USA) were used to treat cells at the time of glucose deficiency. HIF-1α inhibitor (PX-478 and YC-1), PFKFB3 inhibitor (PFK-015) and P-c-Jun inhibitor (SP600125) (MCE, New Jersey, USA) were used separately to test whether LD expression is reduced in relation to the level of glycolysis.

### IFN-γ analysis by enzyme-linked immunosorbent assay (ELISA)

Supernatants were isolated by centrifugation at 1000 rpm for 20 min. The IFN-γ levels in the supernatants were determined using the Human IFN-γ ELISA Kit (Elabscience, Wuhan, China) according to the manufacturer’s instructions. In brief, we added the diluent buffer (100 μL), samples (100 μL) and the standard (100 μL) to wells, incubated for 90 min at 37 °C and removed. After that, we added 100 μL of biotinylated detection antibody for 1 h at 37 °C; immediately, we washed the samples 3 times for 1–2 min each time. Next, we added a horseradish peroxidase (HRP) conjugate (100 μL) to the wells and incubated the plates for 30 min at 37 °C. After washing 5 times, we added the substrate reagent, incubated it for 15 min at 37 °C in dark and then stopped the reaction. We used a microplate reader obtained from Thermo Fisher Scientific to measure the absorbance at 450 nm.

### Western blot analysis

After cell culture for the appointed length of time, total protein was extracted in sodium dodecyl sulfate (SDS) buffer by referring to a previous study^34^, and then the cell lysate was centrifuged at 13,000 rpm for 30 min at 4 °C. The protein concentration of the supernatant was measured using a bicinchoninic acid kit (Thermo Fisher Scientific). A total of 30 µg of protein per lane was separated by 10% SDS-polyacrylamide gel electrophoresis and transferred to a polyvinylidene fluoride membrane (Millipore, Billerica, USA). Then, the membrane was immersed with 5% non-fat milk for 1 h. The membranes were incubated overnight at 4 °C with 1:1000 diluted primary antibodies against PD-L1 (#13684), phosphor-EGFR (#3777), EGFR (#4267), p44/42 MAPK (ERK1/2) (#4695), phosphor-p44/42 MAPK (ERK1/2) (#4370), phosphor-c-Jun (ser-73) (#3270), c-Jun (#9165), LDHA (#3582) and beta-Actin (#4970) (Cell Signalling Technology, Inc., Boston, MA, USA). In addition, the same conditions were used for antibodies against PFKFB3 (ab181861) and HIF-1α alpha (ab16066) (Abcam, Cambridge, England). After washing 3 times for a total 30 min, the membranes were incubated with HRP-conjugated anti-rabbit secondary antibodies (Jackson ImmunoResearch Laboratories, Inc., West Grove, PA, USA; dilution 1:10,000) at room temperature for 2 h. Signals were detected using an enhanced chemiluminescence kit (Millipore, Billerica, USA). The experimental results were normalised to those of β-actin to correct for differences in protein loading. Stripping buffer (Solarbio, Beijing Solarbio Science & Technology Co., Ltd., Beijing, China) was used to elute the antibodies on the PVDF membrane. Densitometric analysis was conducted using Alpha View SA software.

### Reverse transcription real-time quantitative PCR (RT-qPCR)

To quantify PD-L1 mRNA expression, total RNA was extracted using TRIzol (Takara). Then, the total RNA (500 ng) of each group was transcribed into cDNA using a PrimeScript^™^ RT reagent kit (Perfect Real Time) (Takara). All steps were strictly carried out according to the kit instructions and the manufacturer’s recommendations. The following primers were used for the amplification of target mRNAs by RT-PCR. The primers for PD-L1 and β-actin were PD-L1-F: TAT GGT GGT GCC GAC TAC AA; PD-L1-R: TGC TTG TCC AGA TGA CTT GG; β-actin-F: CAG GGC TGC TTT TAA CTC TGG TA; and β-actin-R: CAT GGG TGG AAT CATATT GGA AC. All primers were synthesised by Huada Gene (Beijing, China). qPCR was performed using a Roche LightCycler 480II real-time PCR detection system (Roche, Basel, Switzerland). The relative expression of PD-L1 was normalised to that of β-actin and quantified using the 2−(ΔΔCt) method.

### In vitro proliferation evaluation with an MTT assay and a live-cell imaging system

To evaluate the proliferation of the two cell lines under various in vitro conditions, we followed the common MTT evaluation assay^33^. The MTT assay is based on the conversion of MTT into formazan crystals by living cells, which reflects mitochondrial activity and cell proliferation. 786-O and OS-RC-2 cells were cultivated in a 96-well plate at a density of 2000 cells/well. After culturing for 24 h at 37 °C and 5% CO_2_, the cells were cultured in medium supplemented with a low level of glucose (786-O: 500 mg/L, OS-RC-2: 125 mg/L) and PX-478 (40 μM), YC-1 (10 μM) or PFK-015 (10 μM) for 24 or 48 h. The control group was incubated with fresh RPMI 1640 medium for 24 or 48 h, the same time duration as previous intervention groups. Each sample was evaluated in triplicate in the culture plate. After intervening, 20 μL MTT solution was added to each well, and the plate was incubated further for 4 h at 37 °C. After that, 200 μL of dimethyl sulfoxide was added into each well to terminate the reaction, and then shook by gentle mixing on an orbital shaker for 30 min at room temperature. The optical density was read at 490 nm with a microplate reader (Multimode Varioskan Instrument, Thermo Fisher Scientific). The percentage of cell viability was monitored.

A live-cell analysis system is an analytical system for the long-term dynamic observation of living cells, which can be used to evaluate the proliferative capacity of cells. The two cell lines were seeded in 96-well plates at a density of 2000 cells/well. The cells were evenly adhered to the plate wall by the next day. The medium was replaced with different media for the different intervention groups, including the low-glucose medium group, HIF-1α inhibitor YC-1 group and PFKFB3 inhibitor pfk-015 group, and the 96-well plates were placed in an Incucyte system. The cells were continuously cultured in the cell incubator for 24 h, and the system automatically took photos every 3 h and analysed the cell density of each well. According to the analysis results of the system data, growth curves of the cells from 0 to 24 h were drawn.

### Statistical analysis

Each experiment was performed at least 3 times. All values are presented as mean ± SD and were analysed by analysis of variance (ANOVA) with the statistical software GraphPad Prism 6.0. An unpaired, two-tailed Student’s *t*-test was used to test for significant differences between two experimental groups. The variances between the groups that are being statistically compared were similar. Differences were considered significant at *P* < 0.05.

## Results

### Glucose metabolism regulates cell proliferation

**Fig. 1:**
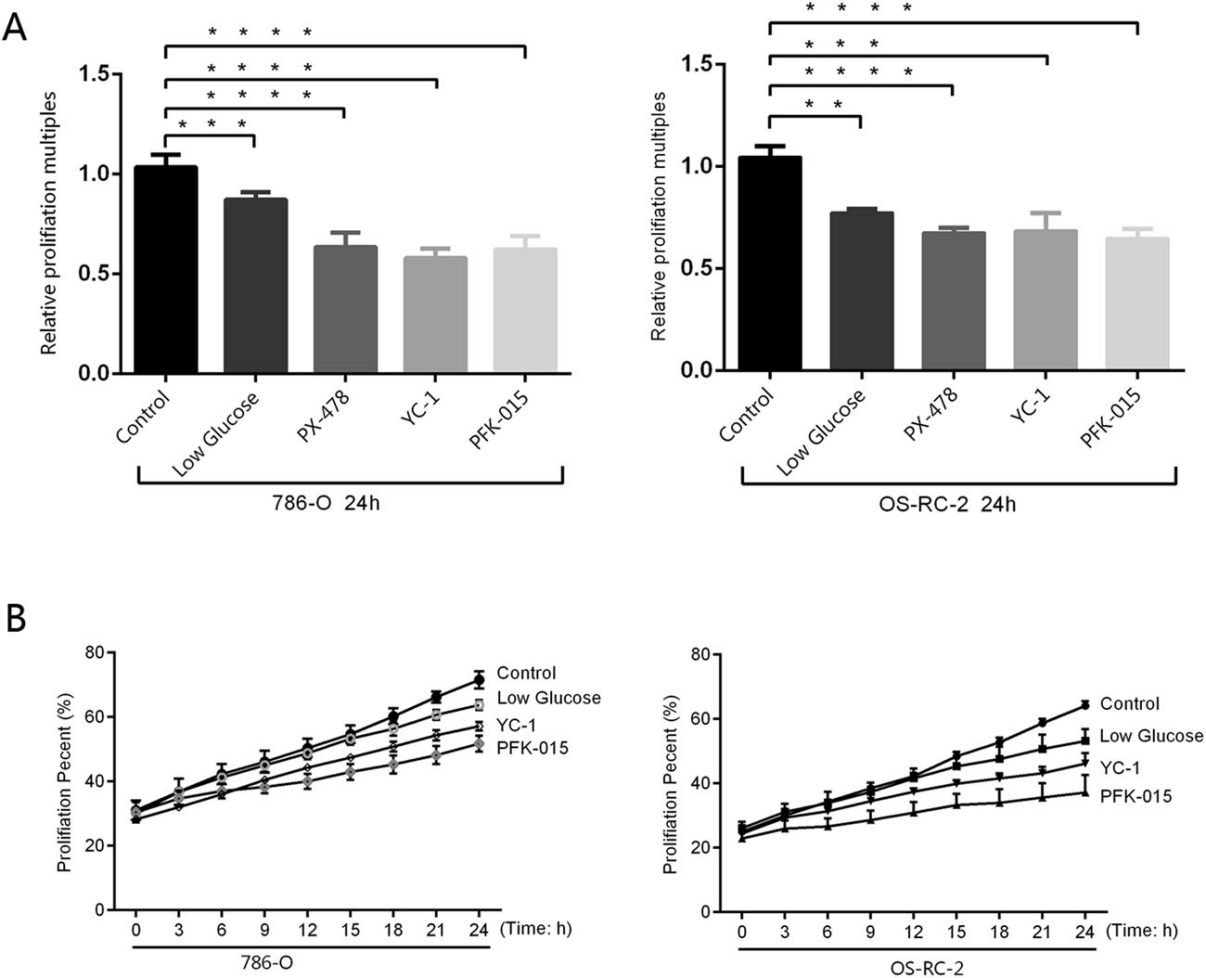
Cell proliferation levels of 786-O and OS-RC-2 cell lines under different cultivation conditions. **A** An MTT assay was used to detect the proliferation of the two cell lines after different interventions. **B** The cell growth curves of the two cell lines under different intervention conditions were observed with a real-time live-cell analysis system. Independent experiments were performed in triplicate. Data are expressed as mean ± SD. ^*^P < 0.05, ^**^P < 0.01, ^***^P < 0.001 and ^****^P < 0.0001.

Two renal carcinoma cell lines, 786-O and OS-RC-2, were cultivated in low glucose concentration medium controlled with normal culture medium for 24 h. The live-cell analysis system and MTT results showed that low glucose concentrations give rise to low proliferation levels (Fig. 1A, B). In the same control group, HIF-1α inhibitors (PX-478 and YC-1) and PFKFB3 (PFK-015) were used to inhibit glycolysis for 24 h. Similarly, the results indicated that the proliferation levels of the three groups, PX-478, YC-1 and PFKFB3, were lower than those of the control group (Fig. 1A). The results from the live-cell analysis system showed that the two intervention groups YC-1 and PFK-015 expressed lower proliferation under the same conditions (Fig. 1B). All these changes in glycolysis downregulated the proliferation capacity. In addition, PD-L1 expression, which correlated with immune evasion, was also detected in the same intervention groups.

### The expression of PD-L1 in renal cancer associated with the level of glucose

**Fig. 2:**
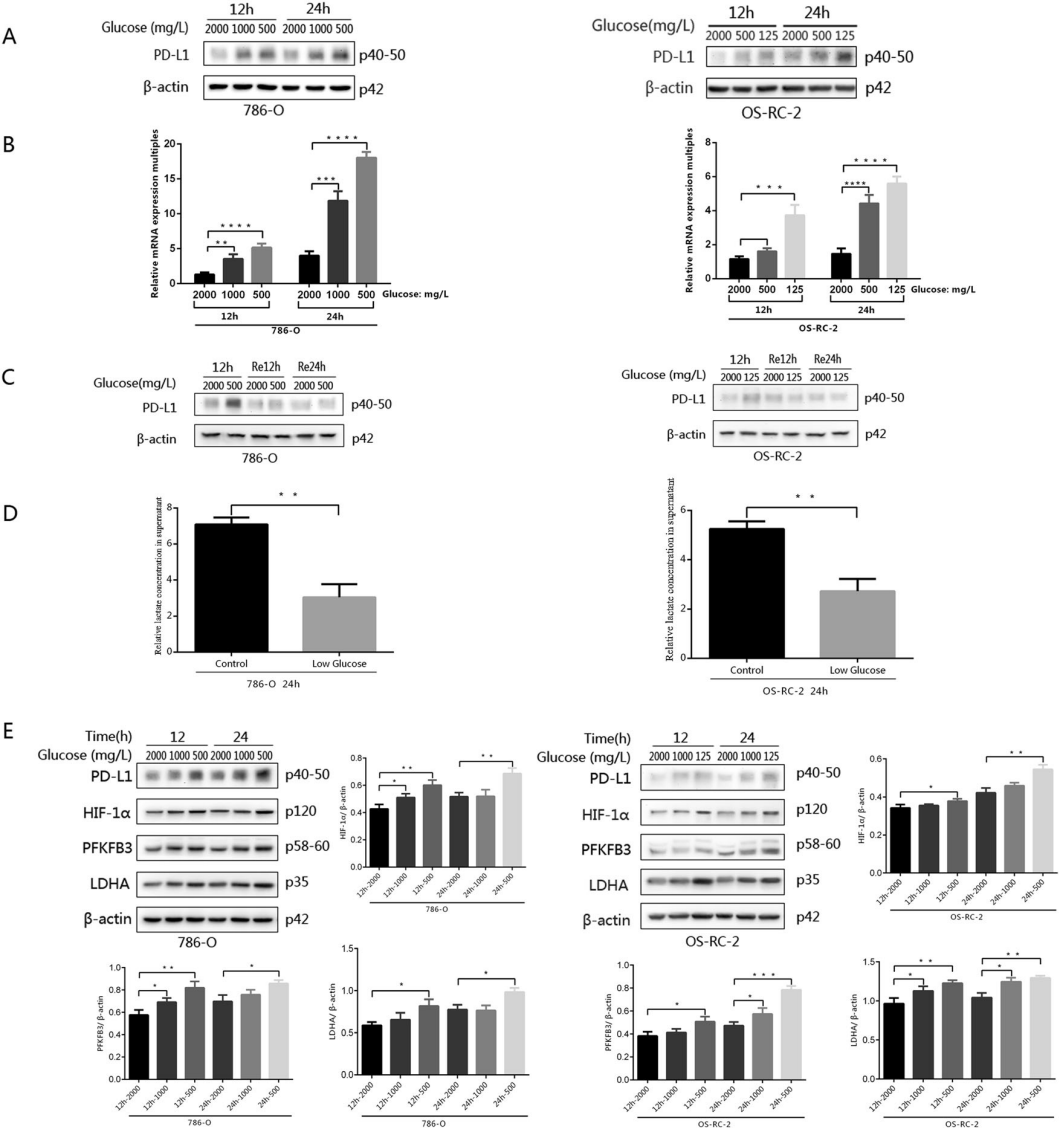
Expression levels of glucose metabolism-related enzymes, products and PD-L1 under different intervention conditions. **A** The expression of PD-L1 in the 786-O and OS-RC-2 cell lines was detected by western blotting after 12 or 24 h of culture under low-glucose conditions. **B** The expression of PD-L1 in the 786-O and OS-RC-2 cell lines was detected by RT-qPCR after 12 or 24 h of culture under low-glucose conditions. **C** Western blotting was used to detect changes in PD-L1 expression in the 786-O and OS-RC-2 cell lines after 12 h of low-glucose culture followed by culture medium replacement with that of normal glucose concentration. **D** Lactic acid (LD) production in the two cell lines were different under low-glucose conditions. **E** Western blotting was used to detect the expression differences in HIF-1α, PFKFB3 and LDHA, key enzymes in glycolysis. Independent experiments were performed in triplicate. Data are expressed as mean ± SD. ^*^P < 0.05, ^**^P < 0.01, ^***^P < 0.001 and ^****^P < 0.0001.

Under glucose deficiency, the proliferation of renal cancer cells declined. However, how do tumours overcome the microenvironment change to regain their lives? It was intriguing to know whether the immune escape function was also changed. PD-L1 expression, which correlated with immune evasion, was detected to analyse the relationship between PD-L1 and glucose. RT-qPCR and western blotting were performed to detect PD-L1 expression in the renal cancer cell lines 786-O and OS-RC-2 with reducing glucose. The results showed that the mRNA level of PD-L1 in the low-glucose group was significantly higher than that in the control with normal glucose (2000 mg/L) treatment, and that PD-L1 expression was increased much more significantly at 24 h than at 12 h, which was consistent with the results of the PD-L1 protein expression experiments (Fig. 2A, B). We also found that the elevated PD-L1 expression induced by glucose deficiency was decreased by glucose recovery (Fig. 2C). In addition, the expression of LD was low under glucose-deficient conditions, which indicated glycolysis downregulation (Fig. 2D). Under the conditions used for PD-L1 detection, the proteins HIF-1α, PFKFB3 and LDHA were tested by western blotting. Their changes in protein levels were the same as those of PD-L1, which was expressed at higher levels with the reduced concentration of glucose (Fig. 2E).

### Glycolysis is reduced by inhibitors of key glycolytic enzymes, giving rise to inverse upregulation of PD-L1

**Fig. 3:**
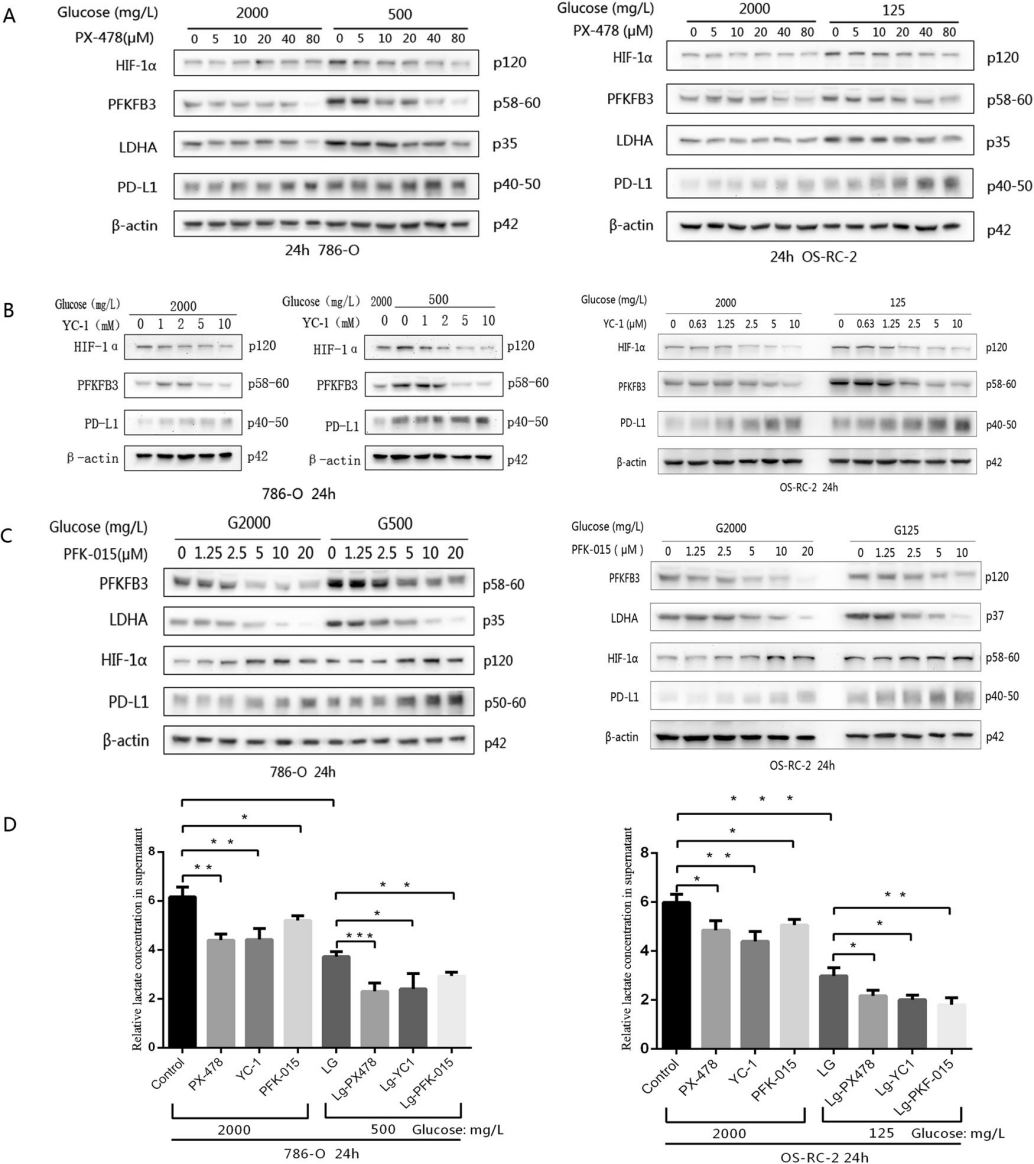
After adding various protein inhibitors to the RPMI 1640 or low-level glucose culture medium system and performing a 24-h incubation, the expression levels of the glycolysis-regulating enzyme HIF-1α, the key enzymes PFKFB3 and LDHA, lactic acid and PD-L1 in the two cell lines were detected by western blotting. **A** After the addition of the HIF-1α inhibitor PX-478 under normal RPMI 1640 or low-glucose culture medium conditions, the expression levels of regulatory enzyme, key enzymes and PD-L1 were detected. **B** Glycolysis-regulating enzyme, key enzyme and PD-L1 expression levels were evaluated in both cell lines after the addition of another HIF-1α inhibitor, YC-1. **C** After the PFKFB3 inhibitor PFK-015 was added, glycolysis-regulating enzyme, key enzyme and PD-L1 expression levels were assessed in the two cell lines. **D** The LD level of two cell lines in RPMI 1640 or low-glucose culture medium treated with the three inhibitors mentioned above is shown. The concentrations of PX-478, YC-1 and PFK-015 were 40 μM, 10 μM and 10 μM, respectively. Independent experiments were performed in triplicate. Data are expressed as mean ± SD. ^*^P < 0.05, ^**^P < 0.01, ^***^P < 0.001 and ^****^P < 0.0001.

As described, HIF-1α genes show very high expression at low glucose concentrations^28^, and the same trend was observed in our glucose-deficiency group. This group exhibited the same expression patterns for PD-L1 and the key glycolytic enzymes PFKFB3 and LDHA after culture with the RPMI 1640 medium culture system for 24 h (Fig. 2E). To investigate the relationship between glycolysis and PD-L1, a HIF-1α inhibitor, PX-478, was added to the intervention group treated with normal RPMI 1640 medium. First, 786-O and OS-RC-2 cells were cultured in the above medium and simultaneously added different concentrations of PX-478 (0, 5, 10, 20, 40 or 80 μM) for 24 h. Both the expression of HIF-1α and that of key enzymes (PFKFB3 and LDHA) decreased in the intervention groups with increasing doses of PX-478, indicating that PFKFB3 and LDHA were downstream of HIF-1α. However, the expression of PD-L1 was increased. Based on the fact that px-478 had a limit to reducing HIF-1α, HIF-1α expression could not be completely blocked as the concentration of PX-478 increased. The elevated level of HIF-1α was obviously suppressed in the low-glucose groups, but an obvious inhibitory effect was not observed in the normal-glucose group (Fig. 3A). The optimal dose of PX-478 that induced the expression of PD-L1 was 40 μM in 786-O cells and 80 μM in OS-RC-2 cells. The level of LD was lower in the 40 µM group than in the group without PX-478 (Fig. 3D). Similarly, PX-478 was also applied in the glucose-deficiency system for 24 h. The level of LD was lower with glucose-deficiency medium than with normal medium (Fig. 3D), and the protein expression of HIF-1α, PFKFB3 and vLDHA was lower with PX-478 treatment in the glucose-deficiency groups, but PD-L1 was highly expressed (Fig. 3A). The results were similar after adding another inhibitor of HIF-1α, YC-1 (Fig. 3B).

An inhibitor of PFKFB3, PFK-015, was also used in the culture system, and the expressions of PFKFB3 and LDHA were obviously inhibited, indicating that LDHA was downstream of PFKFB3, but the levels of HIF-1α and PD-L1 were elevated in both the normal- and low-glucose groups. The LD level was lower with glucose-deficient medium than with normal medium, and it could be dramatically reduced under inhibitor treatment (Fig. 3C, D).

### Low glucose upregulates PD-L1 expression and activates the EGFR signalling pathway

**Fig. 4:**
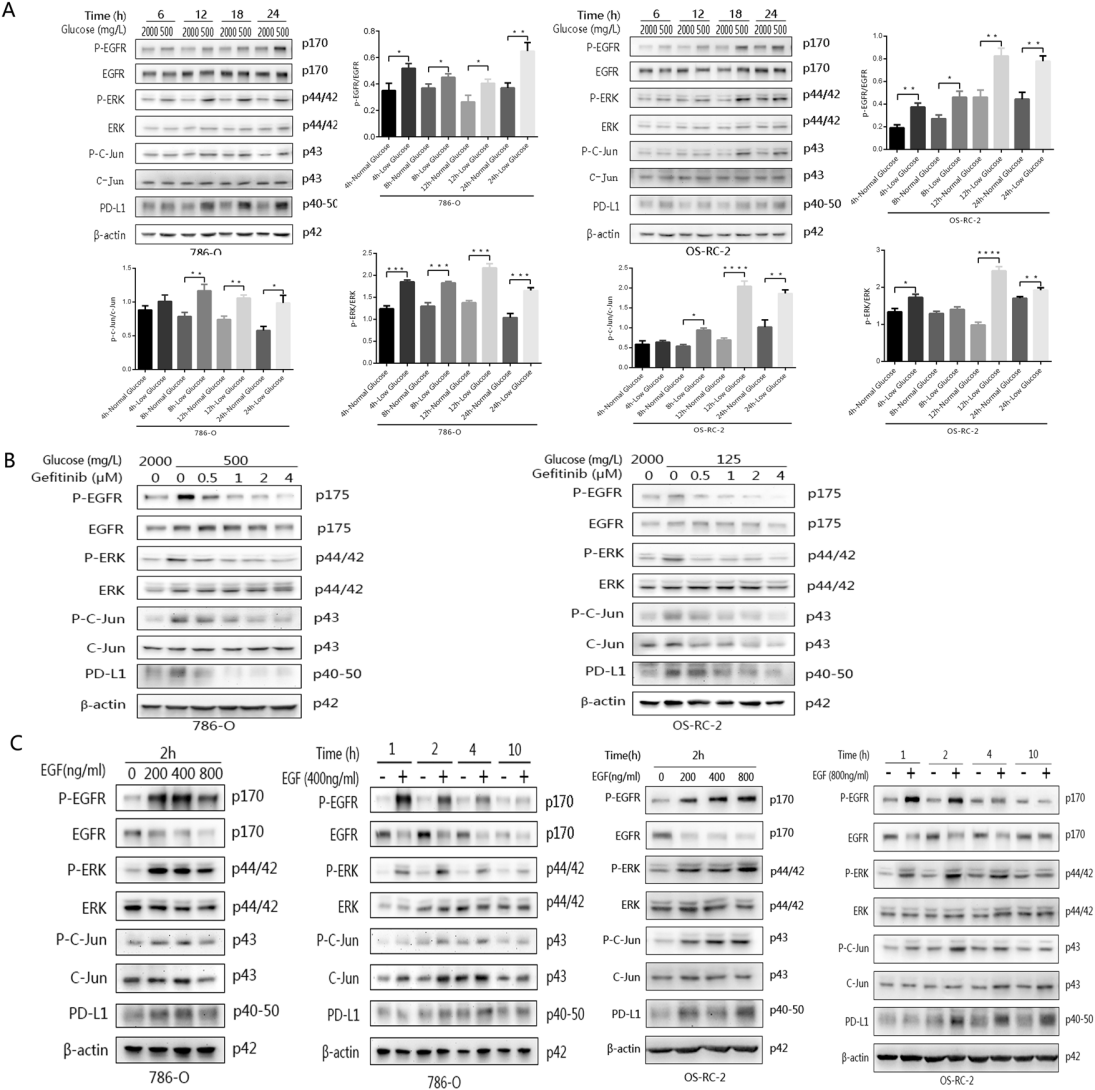
The protein levels of EGFR, ERK, c-Jun and PD-L1 induced in the two cell lines were detected by western blot analysis after treatment with different intervention conditions for 24 h. **A** The expression levels of the various target proteins mentioned above at different time points under low-glucose conditions. **B** The expression levels of the target proteins after the EGFR inhibitor gefitinib was added under low-glucose conditions. **C** The expression levels of the target proteins after the EGFR activator EGF was added under low-glucose conditions. Independent experiments were performed in triplicate.

As glucose metabolism can regulate the expression of EGFR, and the mutation and expression of EGFR can regulate the expression of PD-L1^32^, we hypothesised that PD-L1 expression is regulated by activating EGFR in the context of glucose deficiency in renal cancer cells. To investigate the mechanism by which glucose regulates PD-L1 expression, the EGFR pathway was first examined when glucose was withheld. Renal cancer cells were cultured under normal conditions for 24 h, and the normal medium was then replaced with low-glucose culture medium for 4, 8, 12 or 24 h. The phosphorylation level of the EGFR protein was significantly increased in both cell lines. PD-L1 expression was also dramatically upregulated at 24 h (Fig. 4A). The possibly related proteins ERK1/2 and c-Jun, which are transcription factors and downstream targets of ERK1/2, were also evaluated by western blotting due to the possible involvement of the EGFR/ERK/c-Jun pathway. The results showed that p-ERK and P-c-Jun were apparently upregulated from 4 to 24 h in the low-glucose culture group compared with the normal group, and the differences became increasingly obvious over time (Fig. 4A).

### Elevated PD-L1 expression arising from glucose deficiency is reduced by inhibiting EGFR

To further investigate the regulatory mechanism underlying glucose-mediated activation of EGFR, gefitinib, a first-generation EGFR-TKI, was used. First, the experimental groups were cultured in low-glucose medium and simultaneously treated with different concentrations of gefitinib for 24 h to identify the optimal dose. Then, 786-O and OS-RC-2 cells were cultured with their respective optimal doses for 24 h, and the normal control group and the group undergoing glucose deficiency were compared. The level of P-EGFR was gradually reduced as the concentration of gefitinib increased. At the same time, the protein expression of P-ERK and P-c-Jun gradually decreased in both cell lines (Fig. 4B). Upon adding the optimal dose of gefitinib to the low-glucose culture medium, the upregulation of PD-L1 was reversed after culture for 24 h (Fig. 4B). Overall, these results clearly showed that inhibiting EGFR activation reduced the elevated expression of P-ERK, P-c-Jun and PD-L1 induced by glucose deficiency.

### EGF stimulation enhances elevated PD-L1 expression under glucose deficiency

Since inhibiting EGFR activation downregulated the expression of P-ERK, P-c-Jun and PD-L1 induced by glucose deficiency, EGF, a ligand that activates the EGFR pathway, was used to detect the overlapping effect on the upregulation of PD-L1 expression in the glucose-deficient environment. First, we cultured 786-O and OS-RC-2 cells in glucose-deficient culture medium and simultaneously added different concentrations of EGF (0, 200, 400 or 800 ng/mL) for 2 h. The protein expression of P-EGFR, P-ERK, P-c-Jun and PD-L1 was elevated in both cell lines with increasing doses of EGF. The optimal dose of EGF that induced the expression of PD-L1 was 400 ng/mL in 786-O cells and 800 ng/mL in OS-RC-2 cells (Fig. 4C). Furthermore, 786-O and OS-RC-2 cells were cultured in glucose-deficient culture medium supplemented with 400 ng/mL EGF and 800 ng/mL EGF, respectively, for 1, 2, 4 or 10 h. We found that PD-L1 expression was upregulated from 2 h in 786-O and OS-RC-2 cells (Fig. 4C). PD-L1 expression could be significantly upregulated when glucose deficiency continues over time, and EGF had an overlapping effect on improving PD-L1 expression under these conditions. In summary, these data clearly show that the upregulation of PD-L1 expression is mediated by EGFR activation under glucose-deficient conditions.

### Glucose deficiency upregulates PD-L1 expression through the EGFR/ERK/c-Jun pathway

**Fig. 5:**
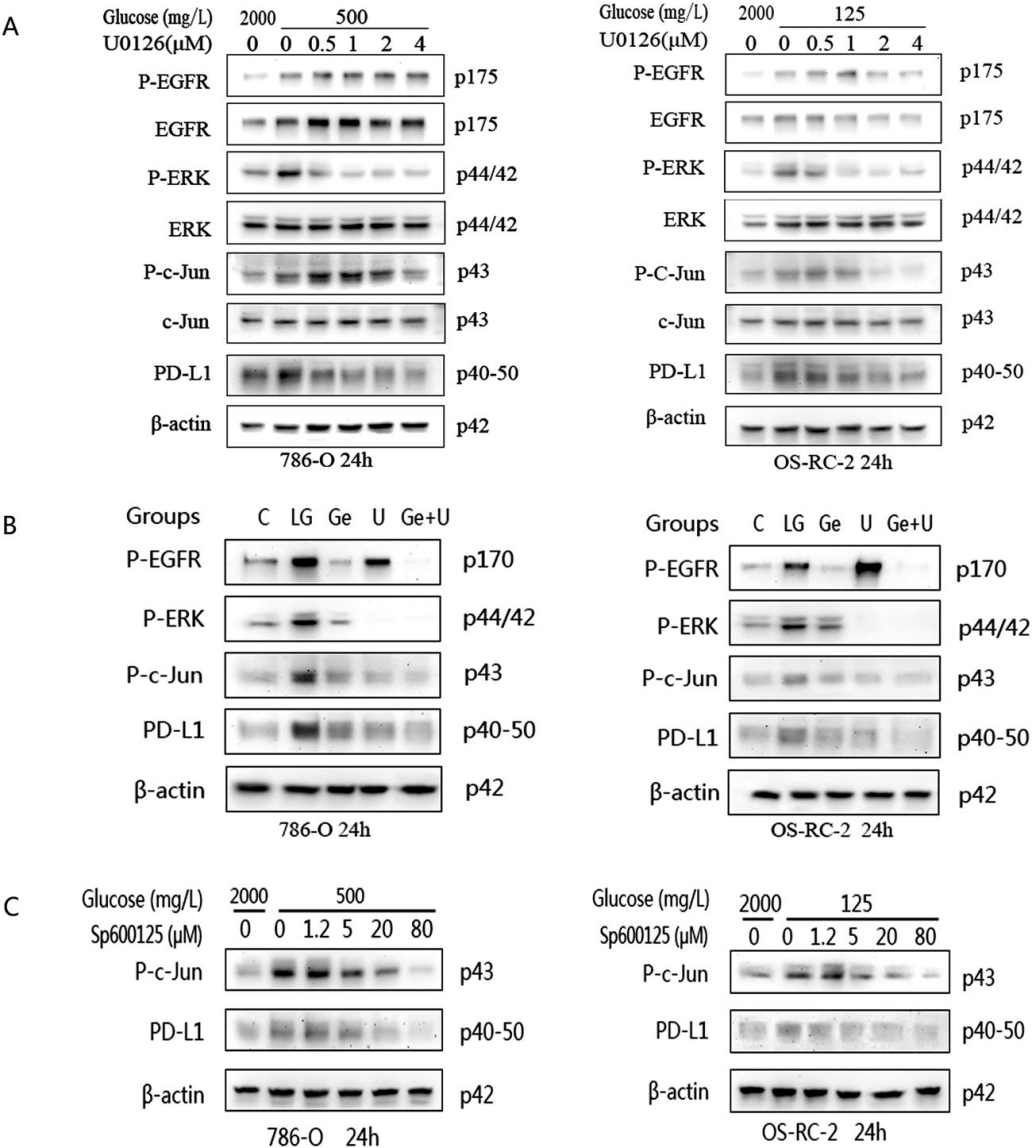
The protein levels of EGFR, ERK, c-Jun and PD-L1 induced in the two cell lines were detected by western blot analysis after treatment with different intervention conditions for 24 h. **A** The expression levels of the target proteins were evaluated after the addition of the ERK inhibitor U0126 during low-level glucose cultivation. **B** After the addition of the EGFR inhibitor gefitinib (Ge), the ERK inhibitor U0126 (U) or the combined application of gefitinib and U0126 (Ge+U), the expression of each target protein was observed. **C** The expression levels of activated c-Jun and PD-L1 were increased by adding the c-Jun inhibitor SP600125 under low-glucose conditions. Independent experiments were performed in triplicate.

ERK1/2 are downstream targets of EGFR, as shown above. To further clarify the involvement of ERK1/2 in the regulation of PD-L1 by glucose, an ERK1/2 inhibitor (U0126) was used to detect the effect of ERK1/2 on the upregulation of PD-L1 expression caused by glucose deficiency. At the same time, c-Jun, a transcription factor and an important downstream target of ERK1/2, was also evaluated. First, 786-O and OS-RC-2 cells were cultured in the low-glucose system with different concentrations of U0126 for 24 h. Control groups were cultured normally for the same time duration. We found that U0126 effectively inhibited the P-ERK1/2 activation induced by glucose deficiency and that the levels of (activated) P-ERK and P-c-Jun gradually decreased as the concentration of U0126 increased. The protein expression of PD-L1 showed a significant reduction at the maximum inhibitory concentration (U0126: 4 μM) (Fig. 5A). To further confirm that upregulated PD-L1 is regulated by EGFR/ERK, the combination of EGFR and ERK inhibitors (gefitinib and U0126) was also applied to the intervention group. The results showed that PD-L1 expression was most obviously inhibited in the combination inhibitor group (Fig. 5B). In addition, to further test whether c-Jun is involved in the regulation of PD-L1 by glucose, an inhibitor of c-Jun, SP600125, was also used to clarify the relationship between c-Jun and PD-L1. After low-glucose cultivation with SP600125 for 24 h, both cell lines expressed P-c-Jun and PD-L1 at lower levels than the corresponding cells cultured without inhibitors (Fig. 5C). In summary, these results show that glucose deficiency upregulates PD-L1 expression through the EGFR/ERK1/2/c-Jun pathway.

### Inhibiting glycolysis under overlapping glucose deficiency to promote PD-L1 expression and activation of the EGFR/ERK/c-Jun pathway

**Fig. 6:**
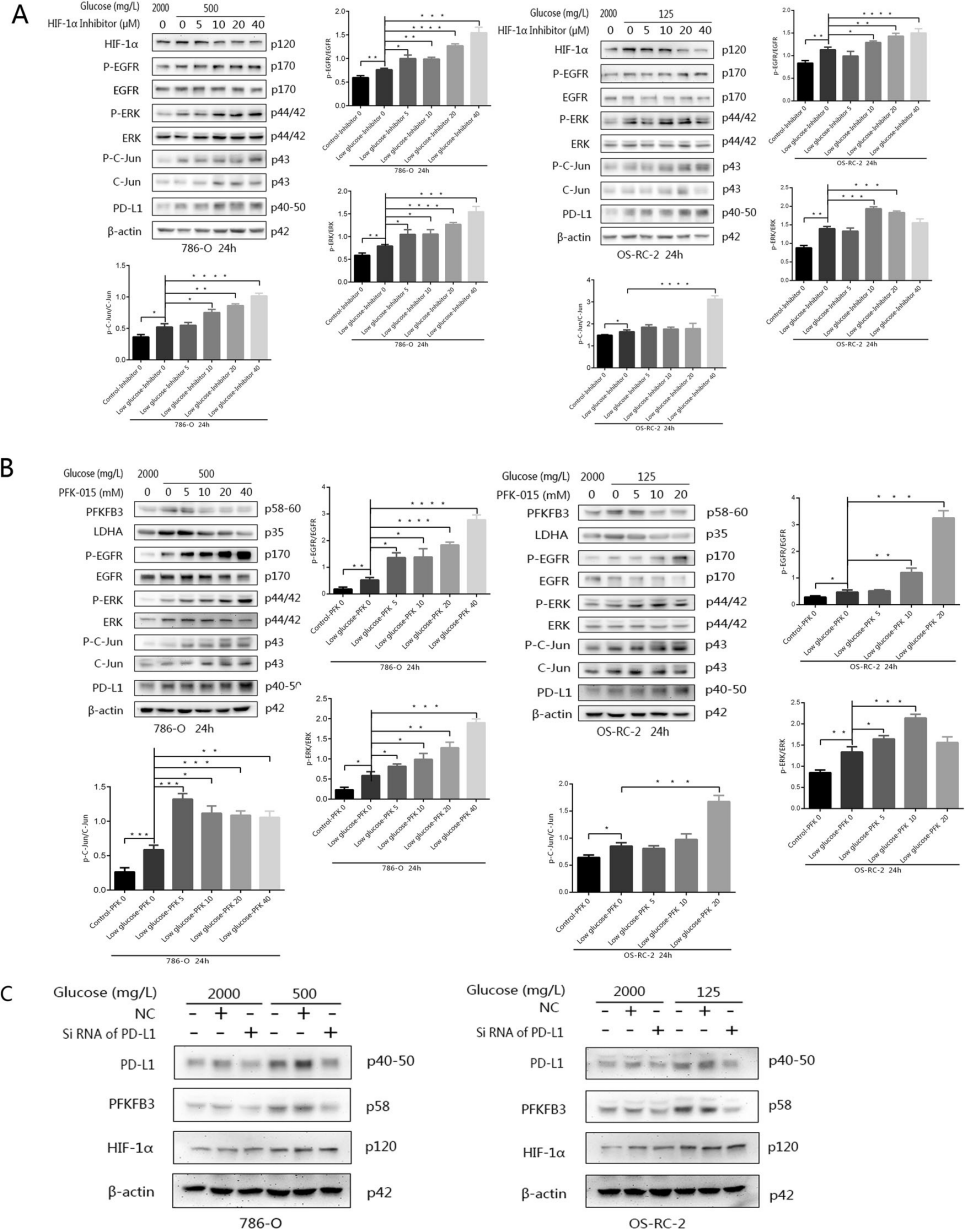
The protein levels of EGFR, ERK, c-Jun and PD-L1 induced by adding different protein inhibitors to the RPMI 1640 or low-glucose culture medium system for 24 h were detected by western blotting. **A** The expression levels of the proteins mentioned above were observed after the addition of the HIF-1α inhibitor YC-1. **B** The expression levels of the proteins mentioned above were observed after the addition of the PFKFB3 inhibitor PFK-015. **C** The expression of PFKFB3, a key enzyme in glycolysis, was observed in both cell lines when PDL1-specific siRNA was added to normal- or low-glucose medium. Independent experiments were performed in triplicate.

To determine whether low glycolysis levels, not only by glucose deficiency, but also by inhibition of key enzymes in glycolysis, can activate this pathway, HIF-1α inhibitor (PX-478) and PFKFB3 inhibitor (PFK-015) were also used to detect the changes in protein expression in the pathway. The results showed that the protein level of P-EGFR was higher after adding PX-478 or PFK-015 to the glucose-deficient conditions than under the same conditions without inhibitors. In addition, activated P-EGFR-related signalling was also activated, and the expression of P-ERK and P-c-Jun was higher in these two groups. The expression of PD-L1 also overlapped to be upregulated in the two cell lines (Fig. 6A, B). Thus, low glycolysis upregulates PD-L1 expression through the EGFR/ERK1/2/c-Jun pathway.

### Reducing the expression of PD-L1 can downregulate the expression of PFKFB3

To explore the relationship between PDL1 and glycolysis, siRNA targeting the PD-L1 was applied. PD-L1 siRNA was transfected by HiPerFect in the normal- or low-glucose culture system for 24 h. Compared with that in the negative control (NC) group, the expression of both PD-L1 and PFKFB3 in the small interfering RNA (siRNA) groups was downregulated. Especially in the low-glucose group, the high expression of PD-L1 was not elevated after knockdown by RNA interference, and the expression of PFKFB3 was also decreased. However, there were no significant changes in HIF-1α expression (Fig. 6C). Therefore, it can be inferred that PD-L1 regulates glycolysis through PFKFB3.

### PD-L1 upregulation deprived from glucose deficiency decreases IFN-γ production by T cells, and PD-L1 increases further and IFN decreases further after glycolysis levels are reduced further

**Fig. 7:**
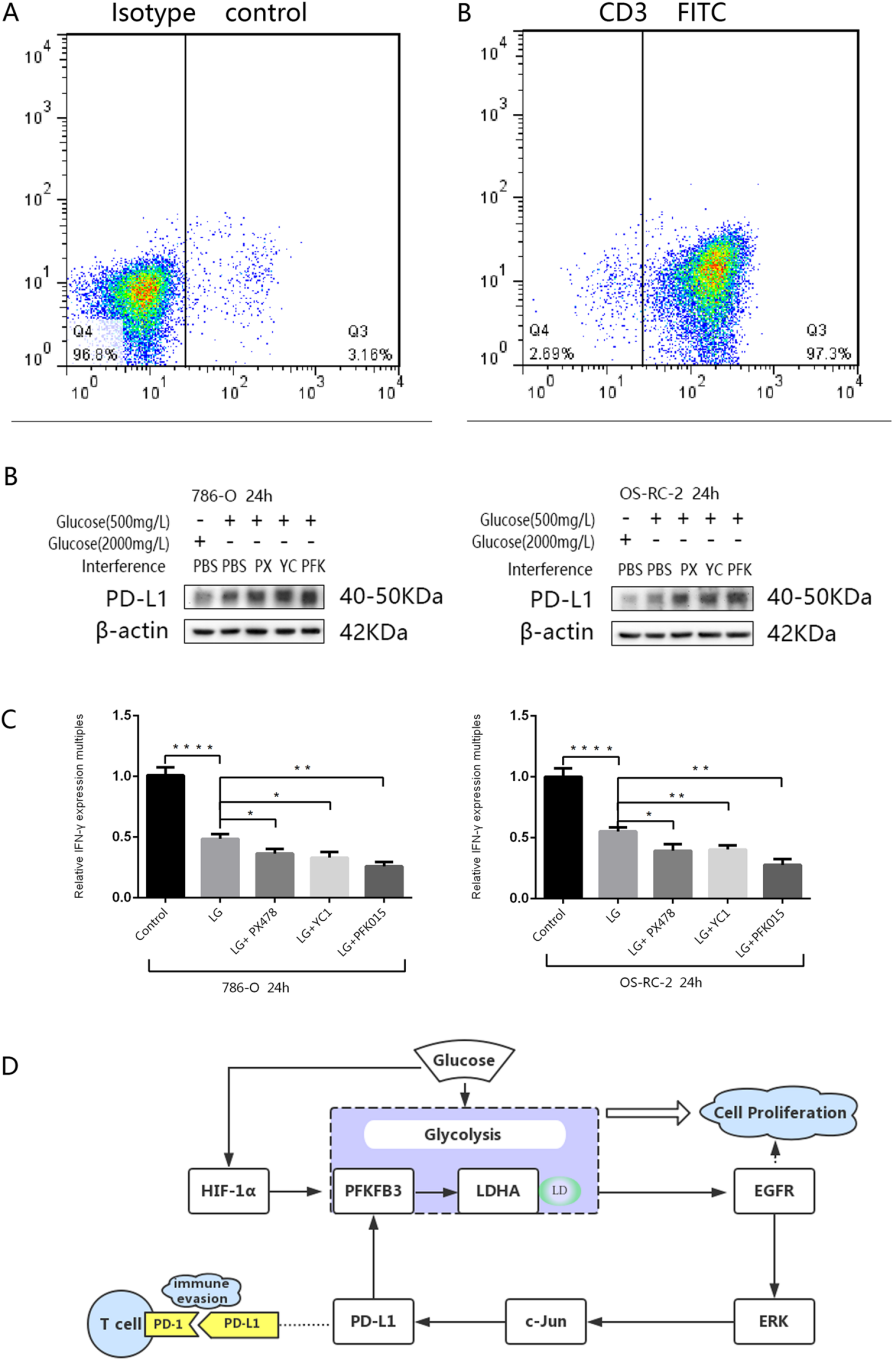
Difference in IFN and PD-L1 expression levels during CD3 and renal cancer cell co-culture under different intervention conditions and integration of the whole mechanism pathway. **A** The purity of T cells was confirmed by flow cytometry using an anti-CD3 FITC-conjugated antibody compared with a mouse isotype control antibody. **B** Protein levels of PD-L1 in 786-O and OS-RC-2 cells cultured in normal culture medium, glucose-free culture medium and glucose-free culture medium with PX-478 (40 mmol/L), YC-1 (10 mmol/L) and PFK-015 (10 mmol/L) for 24 h are shown. **C** After the two tumour cell lines were cultured alone under various conditions for 24 h and then cocultured with CD3+ lymphocytes for another 24 h under normal RPMI 1640 medium conditions, the expression level of IFN-γ in the supernatant of the culture system was detected by ELISA. **D** The pathway diagram summarises the relationship between glycolysis and its associated proteins and PD-L1. Independent experiments were performed in triplicate. Data are expressed as mean ± SD. ^*^P < 0.05, ^**^P < 0.01, ^***^P < 0.001 and ^****^P < 0.0001.

PD-L1 can affect the immune function of T cells, and PD-L1 combined with PD-1 of T cells could mediate immune escape. We investigated whether the upregulated PD-L1 expression during glucose deficiency affected the production of IFN-γ by T cells and whether inhibiting HIF-1α and PFKFB3 facilitated reduction in IFN-γ production. First, CD3+ T cells were prepared from peripheral blood donated by healthy volunteers, and the purity of the cells was confirmed on a BD FACSCalibur after staining with an anti-CD3 FITC-conjugated antibody (Fig. 7A, B). Then, 786-O and OS-RC-2 cells were cultured in glucose-deficient culture medium or normal control medium for 24 h, and the groups undergoing glucose deficiency and simultaneously being treated with PX-478, YC-1 or PFK-015 were compared (Fig. 7C). Then, the culture medium of the above groups was replaced with fresh normal medium, and the cells were cocultured with CD3+ T cells (3*10^5^). After 24 h, 100 ng/mL PMA and 1 g/mL Iono were added to stimulate the cells, for 5 h. The precipitate was removed by centrifugation, and the supernatants were analysed for IFN-γ production. The results showed that IFN-γ production was markedly decreased in the group undergoing glucose deficiency compared with the normal control group and that the production of IFN-γ in the experimental groups treated with YC-1, PX-478 or PFK-015 was lower than that in the group undergoing glucose deficiency (Fig. 7C). Overall, the results showed that the upregulated PD-L1 expression induced by low-level glycolysis could inhibit the production of IFN-γ by T cells.

Based on all of the above data, the relationship between glycolysis and PD-L1 and the relative pathway are listed as a flow diagram (Fig. 7E).

## Discussion

Under the condition of low glucose concentration in the experiment, cell proliferation ability and the glucose metabolism level were decreased; however, the immune checkpoint PD-L1, relating to immune evasion, was improved. In this article, the mechanism underlying how glycolysis regulates and controls the expression of PD-L1 to mediate immune evasion was detected. During the study, the expression of PD-L1 was upregulated in the context of low levels of glucose but recovered to normal after a normal glucose concentration was provided. At the beginning of our experiments, the expression of PD-L1 was found to be increased in the two cell lines, especially the 786-O cell line, with low concentrations of glucose. In addition, 786-O cells were more sensitive than OS-RC-2 cells to glucose deficiency during culture with low-glucose medium, the survival time of 786-O cells was shorter than that of OS-RC-2 cells, and PD-L1 expression increased earlier. OS-RC-2 cells were more tolerant of the low-glucose environment, had a longer survival time and expressed high levels of PD-L1 later. Since PD-L1 levels in these two cell lines were significantly different at the same low-glucose environment, multiple groups of gradient-glucose environment were screened, and those with significant changes were selected. When the glucose concentration was 500 mg/L cultivated for 24 h, the PD-L1 expression of 786-O was improved more significantly than that of the control. At this time, PD-L1 expression changes in OS-RC-2 are still not obvious, and PD-L1 expression is upregulated as the glucose concentration continues to decrease. Therefore, when the glucose concentration dropped to 125 mg/L, PD-L1 expression in OS-RC-2 cells increased significantly.

These two kinds of renal cells were found to exhibit low levels of glycolysis, regardless of whether low levels of glucose were present or key enzymes in glycolysis were inhibited. Then, EGFR was activated to P-EGFR, and a large number of amplified expression levels were obtained, which is consistent with Bollu and Weihua’s study^34^. This process is activated step-by-step, and the downstream protein ERK is activated to P-ERK by P-EGFR^35^ and undergoes a process to induce high expression, which activates c-Jun to produce a high level of P-c-Jun. Therefore, PD-L1 expression is regulated by P-c-Jun^36^. In the low-glucose environment, although HIF-1α, the regulatory enzyme of glycolysis, and PFKFB3 and LDHA, the key enzymes of glycolysis, were highly expressed to try to improve glycolysis, the overall glycolysis level was low due to insufficient levels of the substrate glucose.

EGFR plays a significant role in regulating cell proliferation, survival and migration in normal and cancerous cells^37^. EGFR inhibitors and TKIs have been applied in the clinical treatment of some tumours and have achieved significant therapeutic effects on kidney cancer patients, but some patients are resistant. We hypothesise that these differences are related to immune escape caused by the upregulation of PD-L1 expression. Moreover, EGFR is definitely activated when the level of glycolysis is low^35^. TKI gefitinib inhibited the expression level of EGFR and related downstream proteins, and combined with U0126 inhibiting ERK and downstream proteins, the EGFR/ERK/c-Jun pathway was confirmed to be available, which is consistent with Ikari et al.’s study^38^. To test the upstream/downstream relationship between c-Jun and PD-L1, the c-Jun inhibitor SP600125 was used and showed that PD-L1 was the most downstream protein in the pathway, consistent with the results of one previous study^36^. Therefore, high expression of PD-L1 in renal cancer is mediated by glycolysis through activation of the EGFR/ERK/c-Jun pathway.

Many studies^39,40^ have shown that tumour cells expressing PD-L1 have been shown to transmit inhibitory signals that could increase apoptosis of antigen-specific human T-cell clones and induce differentiation of naive CD4+ T cells into regulatory T cells and maintain regulatory T-cell-suppressive functions by interacting with PD-1, resulting in an abnormal response affecting lymphocyte signal transmission to tumour cells and an inability to kill tumour cells that lead to the occurrence of immune escape.

Furthermore, this study found that when siRNA targeting the PD-L1 was transfected, the PD-L1 level was knocked down significantly. In particular, the high level of elevated PD-L1 induced by low glucose was reduced inversely. When downregulated PD-L1 expression approached its baseline level, PFKFB3 expression was also downregulated, leading to the downregulation of the level of glycolysis. However, the expression of HIF-1α was not affected. The conclusion could be inferred that the mechanism is of practical significance for tumour cells, which need to reduce the level of glycolysis by low PD-L1 expression inhibiting PFKFB3 in the presence of abundant glucose to avoid excessive energy waste and can provide negative feedback to the glycolytic process.

Macroscopically, glycolysis has a significant impact on cell proliferation, metabolism, migration and other life activities. To some extent, glucose supply is insufficient, and glycolysis levels are reduced, which is not conducive to cell proliferation. Therefore, the tumor cells could produce early response. EGFR is phosphorylated and activated, which promotes cell proliferation and regulates the downstream pathway, leading to PD-L1 high expression to prevents the killing effect of lymphocytes. Simultaneously, high expression of glycolysis-related enzymes (such as PFKFB3 and LDHA) attempt to increase the energy production of glucose as much as possible to complete cell proliferation and other life activities. After inhibiting the key enzyme of glycolysis, although the level of glycolysis decreased, which had an inhibitory effect on the life activities requiring energy, such as cell proliferation, it activated the immune escape pathway of tumours.

In conclusion, this study elucidates the effects of glucose metabolism on cell proliferation and the PD-L1-mediated immune escape mechanism in renal cancer cells, which may provide reference for the evaluation of targeted therapy and prognosis of renal cancer as well as the combination therapy of targeted drugs, and to some extent provide theoretical guidance for drug development and clinical medication. However, further animal experiments or clinical sample experiments need to be studied and verified.
