# Correction: Glucose metabolism involved in PD-L1-mediated immune escape in the malignant kidney tumour microenvironment

**DOI:** 10.1038/s41420-023-01652-2

**Published:** 2023-10-19

**Authors:** Yongbo Yu, Ye Liang, Dan Li, Liping Wang, Zhijuan Liang, Yuanbin Chen, Guofeng Ma, Hui Wu, Wei Jiao, Haitao Niu

**Affiliations:** 1 Department of Urology, The Affiliated Hospital of Qingdao University, Qingdao, 266003 China; 2 Key Laboratory, Department of Urology and Andrology, The Affiliated Hospital of Qingdao University, Qingdao, 266003 China

Correction to: *Cell Death Discovery* 10.1038/s41420-021-00401-7, published online 18 January 2021

In Fig. 5A, the images of the ERK protein were used by mistake. We apologize for confusing the WB original figures named ‘2-O786,OSRC-EGFR-24h-U0126-c,pc,0.5,1,2,4’(the band of EGFR) and ‘2-O786,OSRC-ERK-24h-U0126-c,pc,0.5,1,2,4’(the band of ERK), so the band of EGFR was shown twice in Fig. 5A, while the real band of ERK (raw data: figure named ‘2-O786,OSRC-ERK-24h-U0126- c,pc,0.5,1,2,4’) was not shown in this article.

**Figure:**
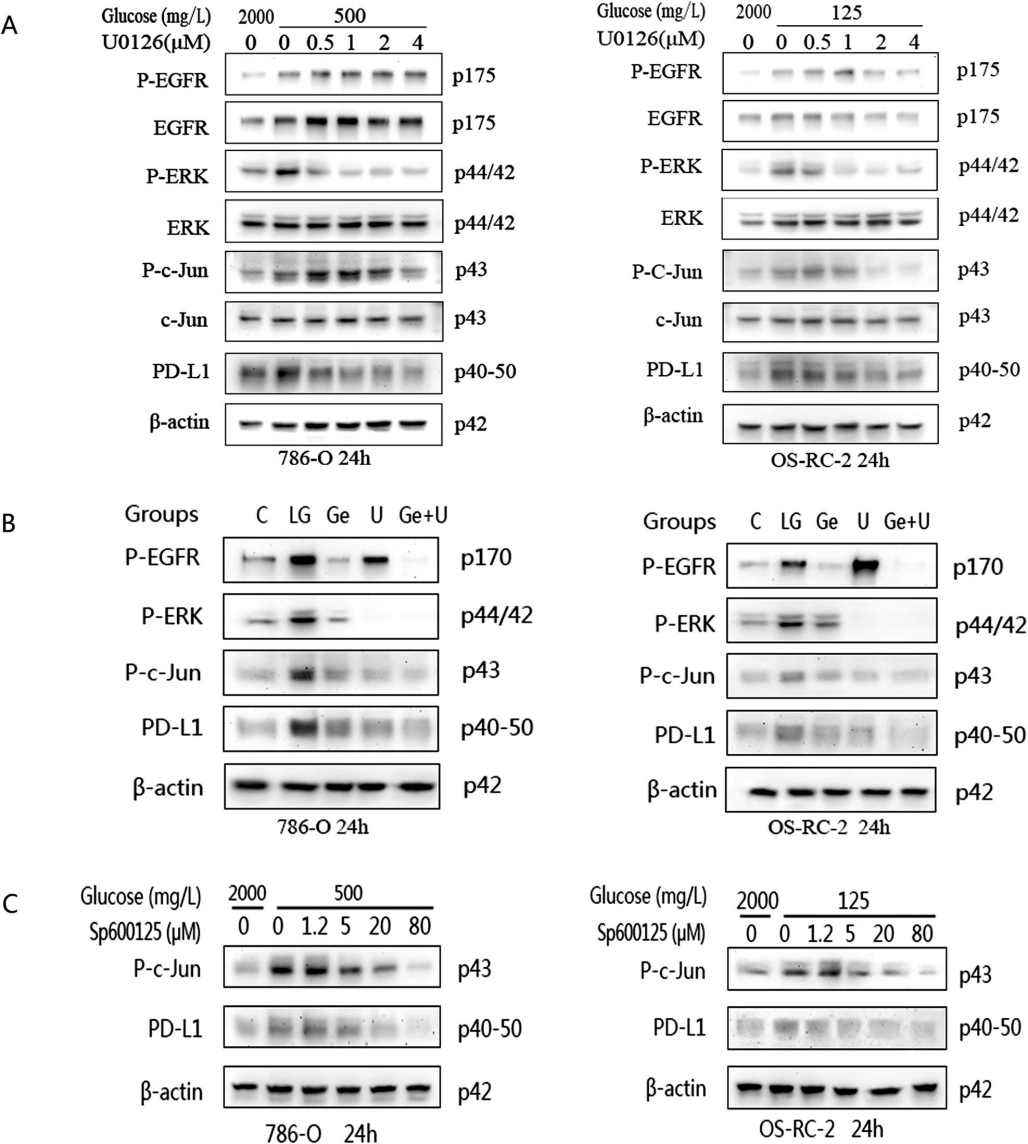

The original article has been corrected.

